# Quinine in Intermittent and Remittent Fevers

**Published:** 1843-05

**Authors:** S. S. Ransom

**Affiliations:** Burlington, Iowa


*Quinine in Intermittent and Remittent Fevers.* By S. S. Ransom, M. D., of Burlington, Iowa.—After several years' practice in the West, where intermittent and remittent fevers prevail, and after watching the medicinal operation of sulphate of quinine upon the system in these diseases, my former views in regard to its operation have become entirely changed. Heretofore I have regarded it as a powerful tonic, and admissible only where there was a perfect intermission (which is, I believe, the generally received opinion). I have, within the last three or four years, given it without regard to the intermission, remission or exacerbation, and with decidedly better success. A remittent very soon becomes intermittent under its use. We will take, for example, a case of *bilious remittent*, which is the most common form of fever in this Valley. After the bowels are thoroughly evacuated with mercurial cathartic, commence with one grain of the s. q. every hour, and it is rarely necessary to continue longer than thirty-six hours before there is a perfect intermission. Before its use, the pulse was from 100 to 120; skin hot and dry; tongue dry; great thirst; violent pain in the head and back. After the system has been kept under its use for a few hours, all these violent symptoms entirely vanish, and a speedy convalescence ensues.

Now what is the medicinal operation of the sulphate quinine on the system? I know of no article in the whole materia medica which produces the same results. It has been accused of producing enlargements of the spleen. I have frequently known it reduce them, and I verily believe that it is among our most valuable remedies in chronic enlargement of that organ.

If you should consider this hasty communication of sufficient consequence, you are at liberty to give it a place in your valuable Journal, for the sole purpose, on my part, of eliciting some light—for it does appear to me that this very valuable article of our materia medica is but imperfectly understood. I would propose that some more able than myself in the profession communicate their views, and I may at some future day give a detailed account of the many cases where I have administered the article, and the results.—*Boston Med. and Surg. Journ.*

